# A Patient-Led Survey of Antibody Drug Conjugate Usage and Dosing for People Living With Metastatic Breast Cancer

**DOI:** 10.1177/23743735261457837

**Published:** 2026-05-30

**Authors:** Amy Beumer, Martha Carlson, Abby L. Arcuri, Julia Maues, Janice Cowden, Stephanie Walker, Kelly Shanahan, Jo Lynn Collins, AJ Veach, Hope S. Rugo, Mark E. Burkard, Kevin M. Kalinsky, Aditya Bardia, Maryam B. Lustberg, Elizabeth Lerner Papautsky, Mya L. Roberson

**Affiliations:** 1 Patient Centered Dosing Initiative, Washington DC, USA; 2 Department of Health Policy and Management, 2331 Gillings School of Global Public Health, University of North Carolina at Chapel Hill, Chapel Hill, NC, USA; 3 20220 City of Hope Cancer Center, Duarte, CA, USA; 4 502602 University of Iowa Holden Comprehensive Cancer Center, Iowa City, IA, USA; 5 Emory Winship Comprehensive Cancer Center, Atlanta, GA, USA; 6 8783 University of California Los Angeles Jonsson Comprehensive Cancer Center, Los Angeles, CA, USA; 7 Yale Cancer Center, New Haven, CT, USA; 8 Biomedical and Health Information Sciences, College of Applied Health Sciences, University of Illinois Chicago, Chicago, IL, USA; 9 Lineberger Comprehensive Cancer Center, Chapel Hill, NC, USA

**Keywords:** metastatic breast cancer, patient engaged research, precision medicine

## Abstract

Scientific advancements have led to more people living longer with metastatic breast cancer (MBC). Antibody-drug conjugates (ADCs) offer a promising treatment option, but currently approved ADCs are associated with significant side effects that can impact quality of life. Dose reductions may help mitigate these effects, yet little is known about real-world, patient-reported experiences with ADC dosing. We surveyed 170 individuals with MBC who had received ADC treatment to assess initial dosing, dose modifications, and barriers to supportive care. Most respondents (94.1%) began treatment with the recommended dosing frequency and 82.9% with the recommended dose. However, 35.3% reported dose reductions, primarily due to experienced or anticipated side effects. Open-ended responses underscored the burden of side effects: “If not for low dose, I don’t think I could go on.” Most participants (81.0%) reported oncologist-led efforts to manage side effects, and 73.0% used medications for symptom control. Financial barriers were common, with 43.5% reporting difficulty accessing supportive care. These findings highlight the need for patient-centered dosing strategies and improved access to supportive care.

## Introduction

Metastatic cancer involves the spread of a primary tumor to distant organs, with common sites in breast cancer (MBC) including the bone, lung, liver, and brain^1^ During the past several decades, survival rates for people with metastatic breast cancer (MBC) have improved due, in part, to an increasing number of effective therapies.^2^ A study comparing 1975 to 2019 estimated that advances in MBC treatment accounted for approximately 30% of the reduction in breast cancer mortality in the United States.^2^

People with MBC typically remain on lifelong treatment, introducing challenges such as persistent toxicities that affect quality of life.^3^ Many therapies cause side effects such as fatigue, gastrointestinal issues, neuropathy, osteoporosis, neutropenia, and cardiotoxicity.^4^ MBC and its treatment can also disrupt employment and increase financial toxicity through medical costs and reduced earning capacity. In 2015, MBC was associated with an estimated $379 million in lost work and home productivity days, an amount likely to have increased since.^5^

Unacceptable toxicities and limited efficacy of traditional chemotherapies have driven research into targeted therapies using subtype, genetic, genomic biomarkers, and new drug delivery methods.^6^ Antibody-drug conjugates (ADCs) are a type of cancer treatment that uses antibodies to bind specifically to cancer cells and deliver chemotherapy, targeting tumors more precisely while attempting to limit damage to healthy cells.^7^ There are currently four FDA-approved ADCs for treatment of MBC across all subtypes, many more in the clinical trial pipeline.^8^

Because of their more targeted mechanism of action compared to traditional chemotherapy, ADCs are often perceived as well-tolerated.^9^ However, their distinct toxicity profiles can lead to cumulative and intolerable side effects that necessitate dose reductions, treatment delays, or discontinuation.^10^ While clinical trials typically adhere to standardized dosing protocols within a well-defined, and generally healthier population, real-world practice is more variable, with dose modifications driven by toxicity, patient preferences, and logistical constraints.^11^ The objective of this study was to conduct a patient-led survey through the Patient Centered Dosing Initiative that elicited and characterized the lived experience of dosing and side effects of ADCs for people with MBC.

## Methods

### Study Design and Participants

This study used a self-administered online survey of patients with MBC who reported being prescribed an ADC. To be eligible, participants were required to self-report having been diagnosed with MBC by a health professional, be at least 18 years old, self-report having taken one of the FDA-approved ADCs for MBC or an ADC in a clinical trial, reside in the United States, and provide informed consent. Recruitment occurred through 21 closed MBC patient advocacy networks, including MBC-focused support groups and Facebook communities. These closed groups consist primarily or exclusively of individuals who self-identify as living with MBC, which increased the likelihood that outreach would reach people who met the eligibility criteria. Members of the Patient Centered Dosing Initiative, all of whom are themselves living with MBC, shared the survey link within these networks. This approach allowed the study team to leverage existing relationships and trust within MBC community spaces and so that invitations came from peers rather than from an academic institution. This survey remained active from February 1, 2025, to March 3, 2025. No protected health information was collected.

### Measures

Survey questions were developed and informally piloted and refined by MBC patient advocates in consultation with oncologists and researchers on the Patient Centered Dosing Initiative Advisory Board. MBC patient advocates reviewed for interpretability while the medical advisory board reviewed the instrument for clinical relevance. The survey collected demographic characteristics including age, gender, sexual orientation, race, ethnicity, and educational attainment. Additional items addressed primary treatment locations, secondary care sites, specialist treatments, type of MBC diagnosis, and the specific ADC received. Response formats consisted of discrete multiple-choice items, select-all-that-apply options, and free-text responses. The estimated completion time was approximately 20 minutes. For each ADC a patient reported receiving, the survey included items assessing starting dose and frequency, any subsequent changes to dose or frequency, experienced side effects, and approaches used to manage side effects. There were in total 103 items (100 multiple-choice and 3 free-text). However, skip logic ensured that participants only viewed questions relevant to the ADCs they personally received. For example, a respondent who reported taking trastuzumab deruxtecan was shown 53 total items, including 50 multiple-choice and 3 free-text questions. This study was deemed exempt by the Advarra Ethics Committee (approval no. Pro00083526) on January 31, 2025. This study qualified for an ethics exemption because it involved anonymous survey data collected from adults, with no identifiable private information and minimal risk to participants.

### Statistical Analysis

Descriptive statistics, including counts and frequencies, were calculated to evaluate the occurrence of ADC dose modifications, such as frequency adjustments or dose changes. Chi-Squared Tests were used to evaluate differences in demographic characteristics among participants who had dose modifications. Additional summary statistics assessed the occurrence of side effects and the use of supportive care measures to mitigate them. Given the exploratory, patient-centered nature of this Research Brief and the goal of characterizing real-world experiences rather than testing pre-specified hypotheses, we used descriptive statistical methods. This approach is well-suited to generating preliminary insights from patient-reported data that can inform future hypothesis-driven research and clinical practice improvements. All analyses were conducted using R.

## Results

In total, 208 respondents initiated the survey. Of those, 11 (5.2%) were ineligible because they did not have MBC or they did not report receiving an ADC, 27 (13.0%) initiated but did not complete the survey, resulting in 170 complete responses (Table 1).

**Table 1.** Patient Characteristics

Patient Characteristic	Overall (n=170)	Not Receiving Dose Reductions (n=101)	Receiving Dose Reductions (n=66)	‘I don’t know’ if received dose reduction (n=3)
**Age (Mean, SD)**	​			
​	53.51 (11.1)	52.19 (10.7)	55.14 (11.4)	62.00 (10.6)
**Gender**	​			
Male	1 (0.6)	1 (1.0)	0 (0.0)	0 (0.0)
Female	169 (99.4)	100 (99.0)	66 (100.0)	3 (100.0)
**Sexual Orientation**	​			
Asexual	4 (2.4)	3 (3.0)	1 (1.6)	0 (0.0)
Bisexual	4 (2.4)	2 (2.0)	2 (3.1)	0 (0.0)
Heterosexual or straight	151 (89.9)	88 (87.1)	60 (93.8)	3 (100.0)
Homosexual or gay or lesbian	1 (0.6)	0 (0.0)	1 (1.6)	0 (0.0)
Prefer not to say	8 (4.8)	8 (7.9)	0 (0.0)	0 (0.0)
**Race** Select all that apply	N= 175	N= 105	N = 67	N = 3
American Indian or Alaska Native	1 (0.6)	0 (0.0)	1 (1.5)	0 (0.0)
Asian	10 (5.7)	10 (9.5)	0 (0.0)	0 (0.0)
Black or African American	11 (6.3)	7 (6.6)	3 (4.5)	1 (33.3)
White	150 (85.7)	85 (81.0)	63 (94.0)	2 (66.6)
Other	1 (0.6)	1 (1.0)	0 (0.0)	0 (0.0)
Prefer not to say	2 (1.1)	2 (1.9)	0 (0.0)	0 (0.0)
**Ethnicity**	​			
Hispanic/Latino	6 (3.5)	5 (5.0)	1 (1.5)	0 (0.0)
Non-Hispanic/Latino	160 (94.1)	92 (91.1)	65 (98.5)	3 (100.0)
Prefer not to answer	4 (2.4)	4 (4.0)	0 (0.0)	0 (0.0)
**Highest level of education completed**	​			
High school graduate, diploma or the equivalent	14 (8.2)	7 (6.9)	7 (10.6)	0 (0.0)
Some college credit, no degree	17 (10.0)	8 (7.9)	9 (13.6)	0 (0.0)
Trade / technical / vocational training	4 (2.4)	2 (2.0)	2 (3.0)	0 (0.0)
Associate degree	15 (8.8)	9 (8.9)	6 (9.1)	0 (0.0)
Bachelor’s degree	53 (31.2)	35 (34.7)	16 (24.2)	2 (66.7)
Postgraduate degree	62 (36.5)	37 (36.6)	24 (36.4)	1 (33.3)
Prefer not to answer	5 (2.9)	3 (3.0)	2 (3.0)	0 (0.0)
**Type of Cancer Care Facility**	​			
Academic or university-based cancer center	64 (37.6)	37 (36.6)	26 (39.4)	1 (33.3)
Community cancer center or clinic	43 (25.3)	24 (23.8)	18 (27.3)	1 (33.3)
Stand-alone private oncology practice	21 (12.4)	14 (13.9)	6 (9.1)	1 (33.3)
Veterans Affairs (VA) hospital or clinic	1 (0.6)	1 (1.0)	0 (0.0)	0 (0.0)
Other hospital or medical center	27 (15.9)	16 (15.8)	11 (16.7)	0 (0.0)
I don’t know	14 (8.2)	9 (8.9)	5 (7.6)	0 (0.0)

Among the 170 respondents, 141 (82.9%) began treatment with a 100% starting dose. The proportion of respondents who started with a lower-than-recommended initial dose varied by antibody-drug conjugate (ADC): 16.7% of those receiving ado-trastuzumab emtansine, 12.9% of those receiving trastuzumab deruxtecan, and 16.2% of those receiving sacituzumab govitecan. Overall, 60 respondents (35.3%) reported experiencing a dose reduction during treatment. The frequency of dose reductions differed by ADC: 33.3% among those on ado-trastuzumab emtansine, 31.2% among those on trastuzumab deruxtecan, and 42.0% among those on sacituzumab govitecan. There was no statistically significant difference in demographic characteristics between respondents who received dose modifications and those who did not. Among the 60 (35.3%) respondents who reported a dose reduction, 51.6% cited a history of adverse side effects as the reason, while 35.0% reported concern about potential side effects. An additional 5.0% indicated other health problems, including migraines and cough, and 15.0% reported other reasons like quality of life or having a relatively low tumor burden and a desire to minimize side effects

**Figure 1.**
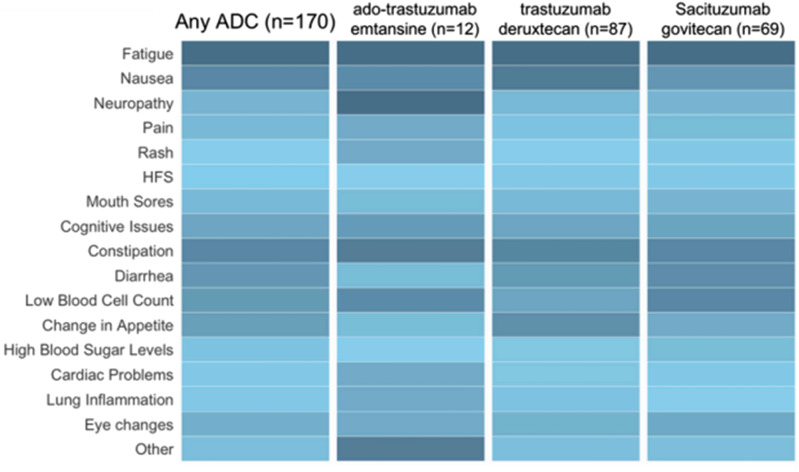
Legend: Darker shades of blue represent a greater number of participants having experienced that side effect, while lighter colors represents fewer participants having that experience

In this study sample, side effects were common, with fatigue (n=131, 77.0%), nausea (n=96, 56.5%), and constipation (n=96, 56.5%) being the most common. Side effect profiles varied considerably by ADC (Figure 1). Most participants (81%) reported that their oncologist took some action to mitigate side effects. A total of 74 (43.5%) respondents reported experiencing financial barriers to accessing supportive care. Among these individuals, 8.1% faced financial barriers to obtaining medication for low white blood cell counts, 5.4% had difficulty affording anti-diarrheal medications and 56.8% experienced financial barriers to other supportive care needs.

## Discussion

This patient-led study among a convenience sample of people living with MBC who self-reported taking an ADC provides real-world evidence that dose modifications are common and often necessary to preserve quality of life, information that is inconsistently captured in clinical trials. By eliciting patient-reported experiences with dosing and side effects not previously described, the survey highlights the frequency of dosing modifications, both dose-level reductions and changes in treatment frequency. As this therapeutic class becomes increasingly widely used, including earlier use in MBC treatment and new use in early-stage breast cancer, the systematic collection and reporting of real-world patient-reported outcomes will be critical for informing strategies to support quality of life and shared decision-making.

Recent pooled estimates of adverse events from sacituzumab govitecan clinical trials indicate the occurrence of side effects as 61% experiencing neutropenia, 64% experiencing diarrhea, and experiencing 51% fatigue.^12^ Though not directly comparable, respondents in our study who had taken Sacituzumab govitecan reported low blood counts and diarrhea at lower frequencies, and fatigue at higher frequencies. Similarly, in a meta-analysis of clinical trials, pooled incidence for trastuzumab deruxtecan was 37.0% for neutropenia, 71.0% for nausea, and 41.3% for fatigue.^13^ Likewise, although estimates are not directly comparable given the self-report nature and convenience sample, respondents in our study on trastuzumab deruxtecan reported all three of these side effects at higher frequencies than reported in trials.

Although ADCs are reported to have a more tolerable side effect profile than traditional chemotherapy, our findings, and those of other recent studies, demonstrate the substantial side effect burden associated with these therapies.^10,14^ Previous Patient Centered Dosing Initiative research has shown that actual side effects or anxiety surrounding expected side effects can be mediated by individualized dosing.^15^ In this convenience sample of people with MBC who self-reported taking an ADC, 81% of participants reported that their oncologist took steps to mitigate side effects, free-text responses revealed that dose adjustments played a role in this management approach. Because individualized dosing may improve quality of life without sacrificing efficacy, supporting shared decision-making in oncology care teams is critical. Shared-Decision making often involve differing perspectives as well as inherent power and knowledge imbalances.^15^ The Patient Centered Dosing Initiative has developed a conversation starter tool on this topic, designed to educate all patients and oncologists and promote Shared Decision making.^15-17^

Real-world patient experiences with ADCs also have important implications for ongoing oncology policy reforms and dose optimization initiatives in the United States.^18,19^ As financial barriers and treatment tolerability become key factors in patients’ ability to stay on therapy, patient-reported data like those collected in this study provide a vital complement to clinical trial evidence, which often underrepresents the total burden of side effects, dose reductions, and supportive care challenges experienced during treatment. ^15-17^ Our findings highlight gaps in dosing frameworks and the need for post-approval evidence, as they document patient experiences with dose modifications and how financial barriers may limit access to supportive care. Integrating patient-reported outcomes into clinical guidelines and regulatory science policy can help ensure that ADC dosing strategies balance efficacy with quality-of-life considerations, ultimately promoting a more patient-centered approach to metastatic breast cancer treatment.

The U.S. Food and Drug Administration (FDA) has begun challenging traditional oncology dosing norms through initiatives aimed at making therapeutic advancements more responsive to real-world patient needs.^19^ The FDA Oncology Center of Excellence’s Project Optimus seeks to shift the focus of oncology therapeutics to dose optimization from the traditional Maximum Tolerated Dose model.^18^ Importantly, the establishment of dosing regimens for the currently FDA approved ADCs occurred prior the establishment of Project Optimus, underscoring a clear need to revisit in light of new priorities in precision patient-centered oncology. In July 2025, in partnership with the American Society of Clinical Oncology, the FDA issued a statement emphasizing the urgency of this shift, outlining five scientific principles for patient-centered dose optimization, including the need to gather and integrate more patient safety and tolerability data.^20^ As these regulatory initiatives continue to evolve, future policy efforts should place greater emphasis on incorporating real-world patient experiences into dosing standards, helping ensure that forthcoming ADC development and approval pathways more fully reflect the lived realities and tolerability needs of people with MBC.

### Limitations

This study has limitations, including a self-selected sample from online advocacy networks, which does not represent all patients receiving ADCs. The sample might overrepresent more engaged or digitally connected individuals. Self-reported data can be biased and lack detailed clinical information. Despite this, the study offers valuable insights into patient experiences with ADCs and highlights areas for future research and improvements in cancer care.

## Conclusions

These findings highlight the complexity of real-world treatment experiences among individuals receiving ADCs for MBC. They underscore the need for more patient-centered approaches to dosing and supportive care in MBC treatment. As the use of ADCs expands, integrating real-world patient experiences into clinical practice and drug development will be essential to optimizing outcomes and enhancing quality of life.

## Data Availability

The data that supports the findings of this study are available from the corresponding author upon reasonable request.*
